# Auer rods in mature granulocytes in peripheral blood

**DOI:** 10.1007/s12185-023-03694-9

**Published:** 2024-01-04

**Authors:** Enrico Schalk, Antje-Friederike Pelz

**Affiliations:** 1 Department of Hematology and Oncology, Medical Faculty, Otto von Guericke University Magdeburg, Leipziger Str. 44, 39120 Magdeburg, Germany; 2 Institute of Human Genetics, Medical Faculty, Otto von Guericke University Magdeburg, Magdeburg, Germany

**Keywords:** Acute myeloid leukemia, Auer rods, Granulocytes, Peripheral blood, inv(16)

A 55-year-old man with agranulocytosis was diagnosed with acute myeloid leukemia (AML). Atypical eosinophil granulocytes with large basophil but also small pale, structureless granules were observed, and cells with Auer rods in maturing granulopoiesis were noticed in the bone marrow. Panel A shows a mature granulocyte in peripheral blood with many Auer rods in cytoplasm (Pappenheim stained peripheral blood smear, 1000x; Supplementary Material). Auer rods were visible in the peripheral blood in 8% (4/50) of the mature granulocytes. Panel B shows fluorescence-*in-situ*-hybridization with inversion 16 [inv(16)(p13q22)] in a bone marrow blast due to rearrangement (split; red and green) of a CBFB copy (1000x) (Fig. 1).

**Fig 1.**
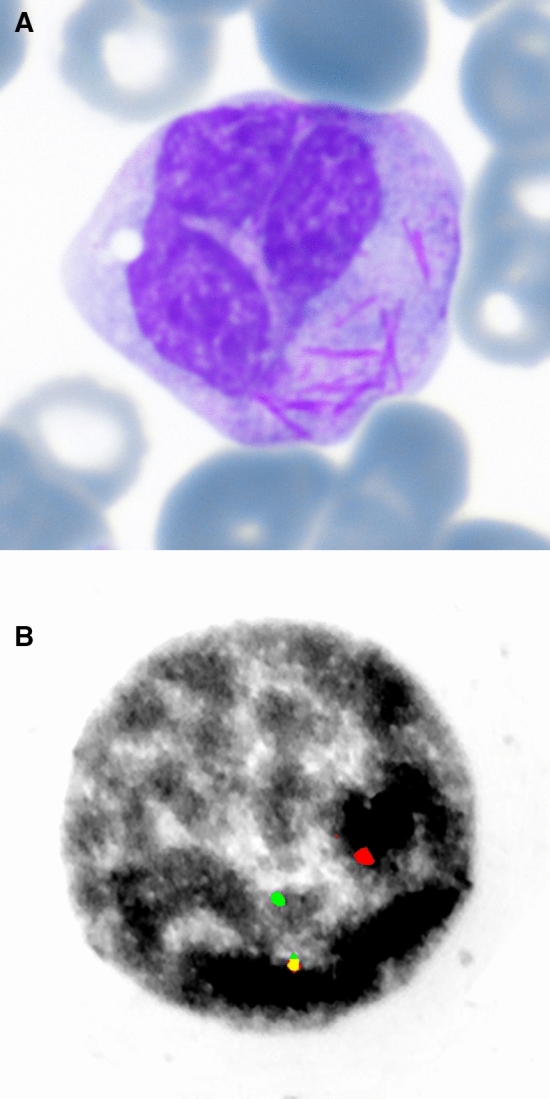
**A** Mature granulocyte in peripheral blood with many Auer rods in cytoplasm (Pappenheim stain, 1000x). **B** Fluorescence-in-situ-hybridization with inversion 16 [inv(16)(p13q22)] in a bone marrow blast (split; red and green; 1000x)

In bone marrow, atypical (“dirty”) eosinophils with Auer rods in maturing granulopoiesis are suspect for AML with inv(16). Auer rods are not found in mature/physiological granulocytes. However, in bone marrow, Auer rods in maturing granulopoiesis are typical for core-binding-factor AML, like AML with inv(16), and for acute promyelocytic leukemia (APL) with translocation t(15;17). They are part of the malignant clone and an expression of granulocytic differentiation of AML blasts normally arrested in maturation. Therefore, in APL patients with specific/targeted (“*in-vivo*-differentiation”) treatment, namely, all-trans-retinoic acid or arsenic trioxide, Auer rods are sometimes found in mature granulocytes in peripheral blood.

However, Auer rods in mature granulocytes in peripheral blood are very uncommon in cases of AML with inv(16), as is spontaneous granulocytic differentiation without “in-vivo-differentiation” therapy.

### Supplementary Information

Below is the link to the electronic supplementary material.

Supplementary file1 (PDF 199 KB)

## Data Availability

Not applicable.

