# Supplementary material for: Auer rods in mature granulocytes in peripheral blood
Source: Int J Hematol. 2024 Jan 4;119(2):105–6. doi: 10.1007/s12185-023-03694-9 (PMC10830820; doi:10.1007/s12185-023-03694-9)
Supplement: Supplementary file 1 — Supplementary file1 (PDF 199 KB) [file 12185_2023_3694_MOESM1_ESM.pdf]

Supplementary Material

Auer rods in mature granulocytes in peripheral blood

Enrico Schalk[1], Antje-Friederike Pelz[2]

[1]Department of Hematology and Oncology, Medical Faculty, Otto von Guericke University Magdeburg, Magdeburg, Germany
[2]Institute of Human Genetics, Medical Faculty, Otto von Guericke University Magdeburg, Magdeburg, Germany

Supplementary Figure 1, Panel A and B

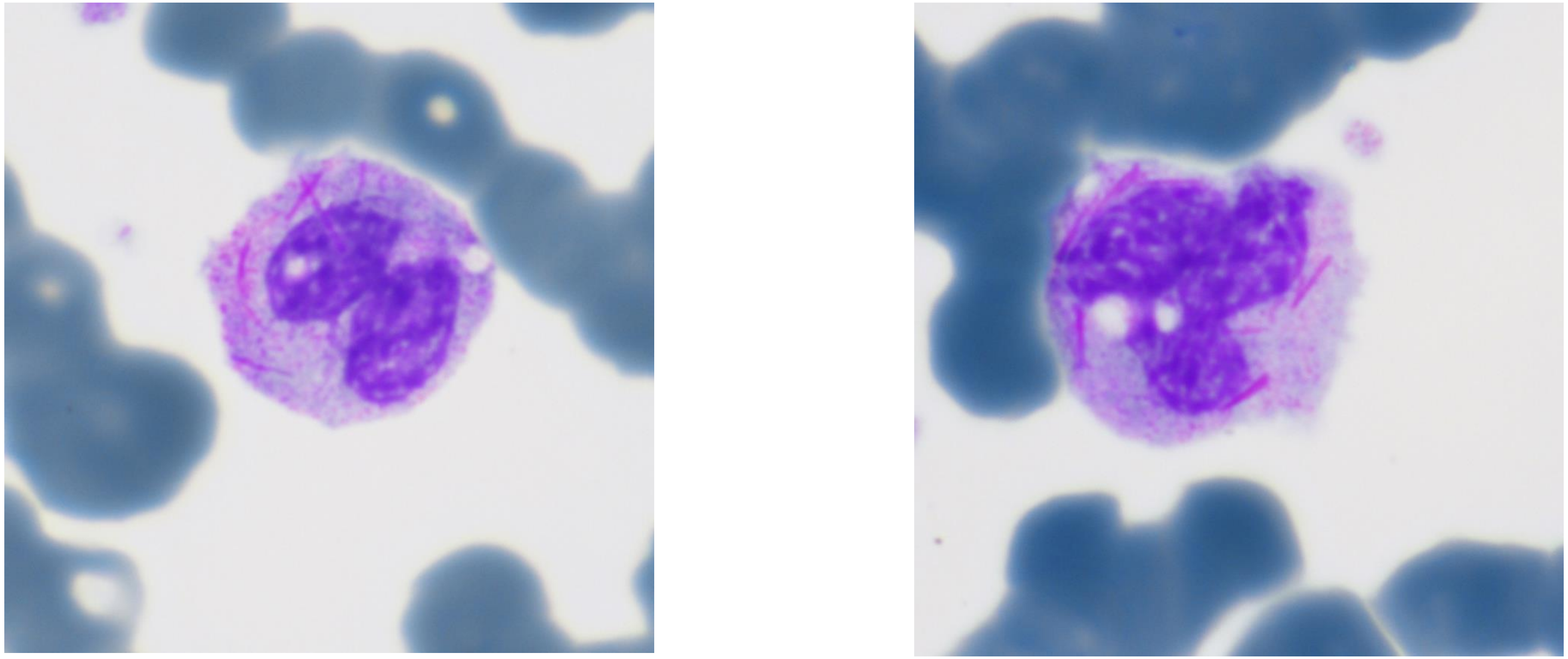

Mature granulocytes in peripheral blood with multiple Auer rods in cytoplasm.
Pappenheim stained peripheral blood smear, 1000x.

Supplementary Figure 2, Panel A and B

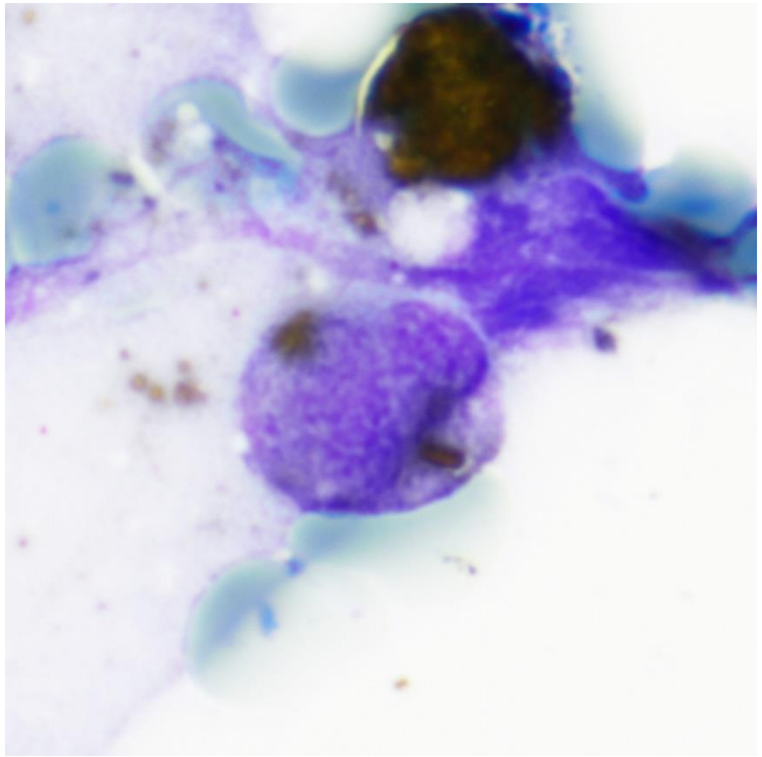

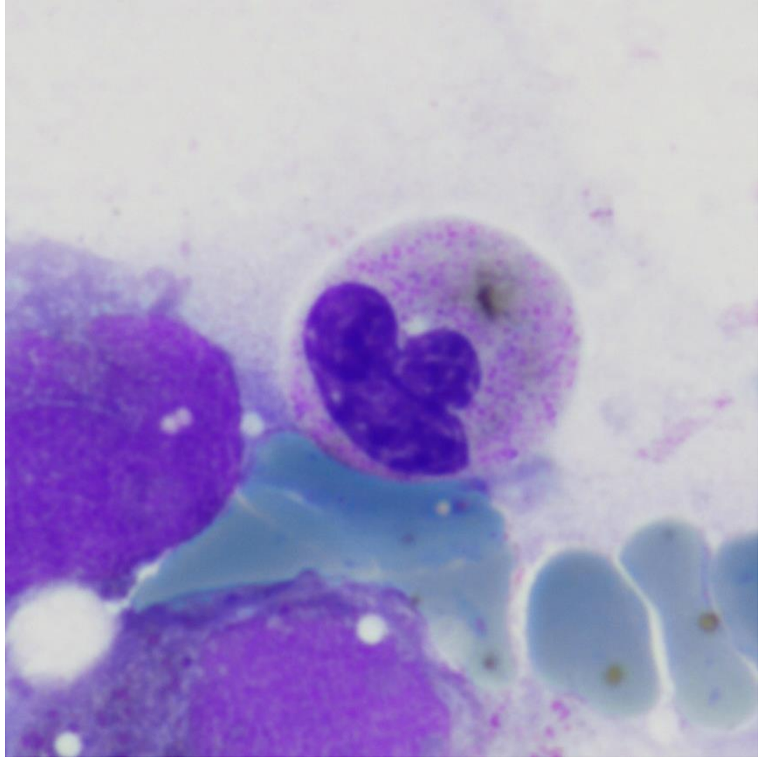

More mature cells of granulopoiesis in bone marrow with Auer bodies/Phi body in cytoplasm.
Myeloperoxidase stained bone marrow smear, 1000x.
